# Supplementary material for: Feasibility of Polyclonal Avian Immunoglobulins (IgY) as Prophylaxis against Human Norovirus Infection
Source: Viruses. 2022 Oct 27;14(11):2371. doi: 10.3390/v14112371 (PMC9698945; doi:10.3390/v14112371)
Supplement: Supplementary file 1 [file viruses-14-02371-s001.zip › viruses-1924499-supplementary.pdf]

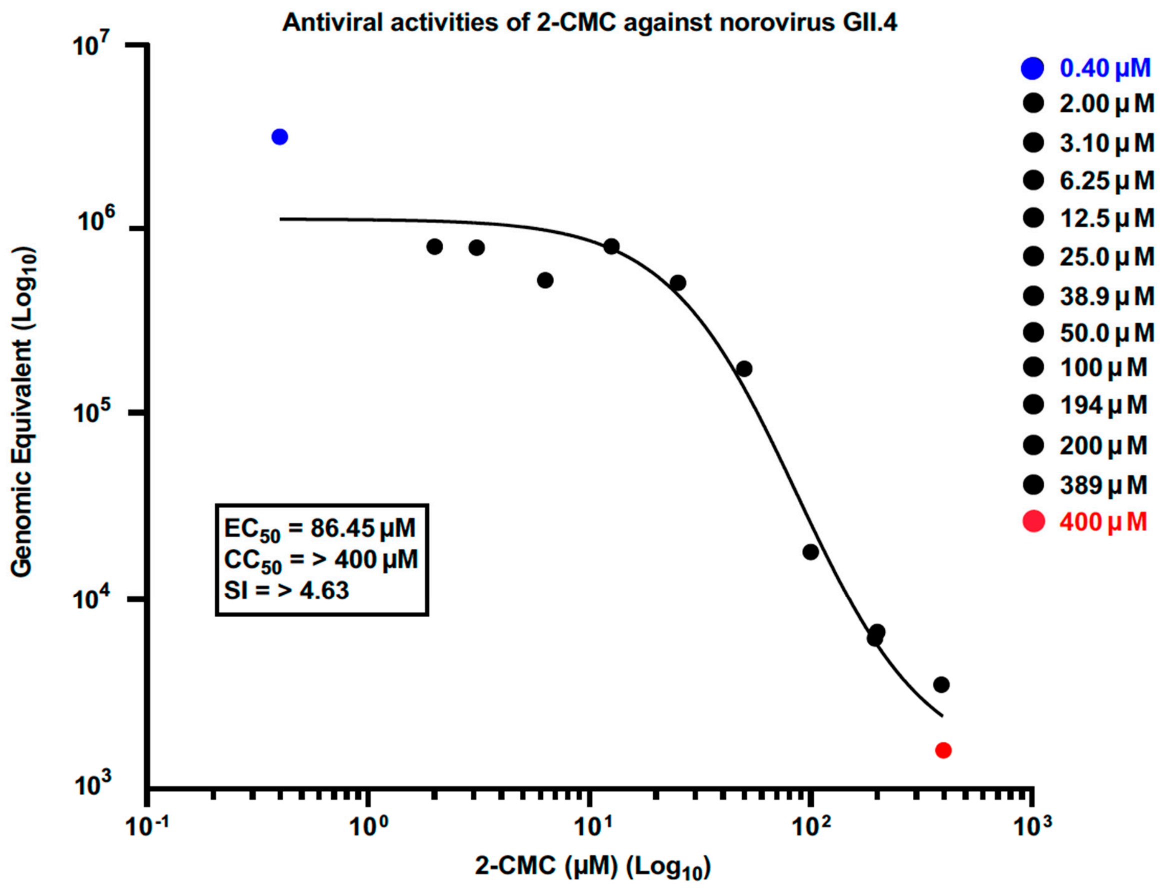

Figure S1. Anti-norovirus activities of 2-CMC against HuNoV GII.4 on HIE J2 cells. 2-CMC: 2'-C-methylcytidine, tested at a concentration range of 0.40 μM – 400 μM. EC_{50}: 50% inhibition concentration; CC50: 50% cytotoxic concentration; SI: selectivity index presented as CC_{50}/EC_{50}.

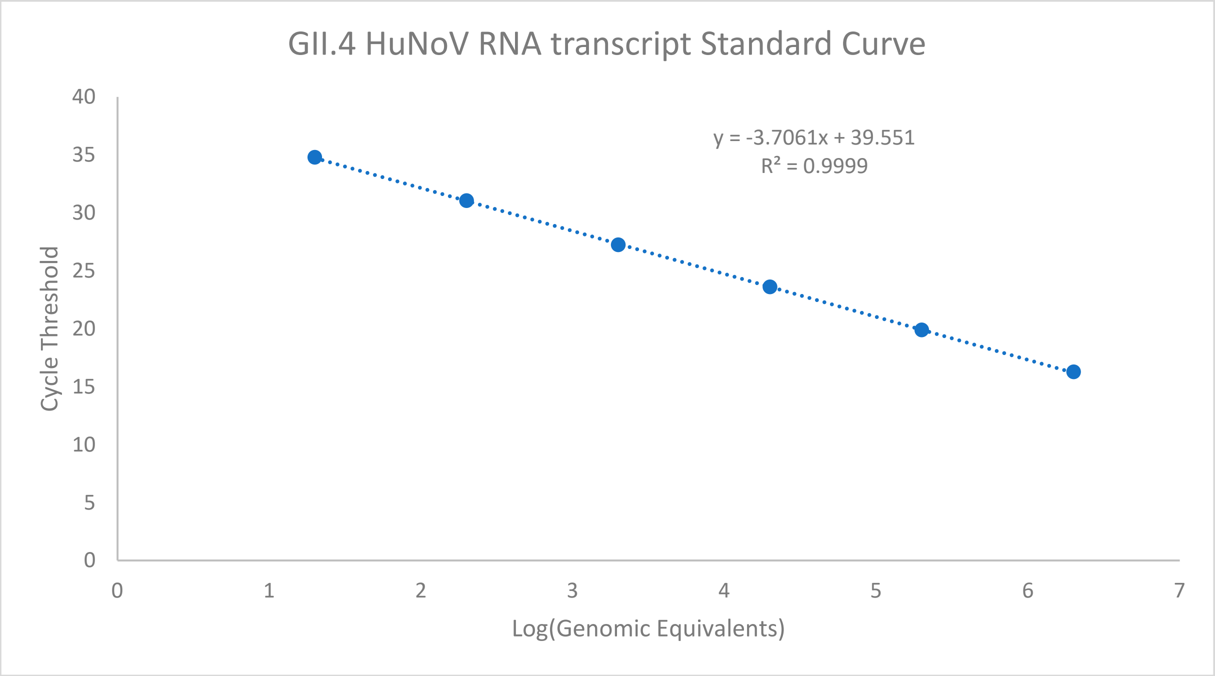

Figure S2. HuNoV RNA transcript standard curve.

The GII RNA transcript was generated following the method of Schwab K.J. et al 1997* with the following modifications. The sequence between nucleotides 3839 and 5511 from GII.4 Houston virus (Hu/Houston/TCH186/2002/US; GenBank accession no. EU310927) was synthesized (Thermo-fisher GeneArt, USA) with a 5' flanking Asc I restriction site – T7 phage polymerase promoter region and a 3' SP6 polymerase promoter – Xho I flanking region. This synthetic insert was cloned into a pMA-7 (AmpR) vector (Thermo-fisher GeneArt, USA), heat-shock transformed into chemically competent E.coli TOP 10 (Thermo-fisher Invitrogen, USA), and then grown on 100 µg/mL Ampicillin selection LB plates for 17 hours at 37°C. Two growing colonies were picked for propagation in liquid selection culture to produce insert containing glycerol bacterial stocks. Sequence analysis on extracted propagated DNA plasmid verified sequence integrity. In vitro transcription was performed with linearized plasmid samples, using the T7 MEGAscript kit (Thermo-Fisher, USA) system with a 2 hour incubation followed by DNA template degradation with a TURBO DNase treatment. The high yield reaction products were purified using the MEGAClear (Thermo-Fisher, USA) clean-up kit to give high purity RNA. GII RNA transcript standard was brought to a final concentration of $2x10^{10}$ genomic equivalents/µL in 5 µL aliquots with RNA Storage Solution (Thermo-Fisher, USA) and stored at -80°C.

*Schwab, K. J., M. K. Estes, et al. (1997). "*Use of heat release and an internal RNA standard control in reverse transcription-PCR detection of Norwalk virus from stool samples*." J Clin Microbiol **35**(2): 511-4.

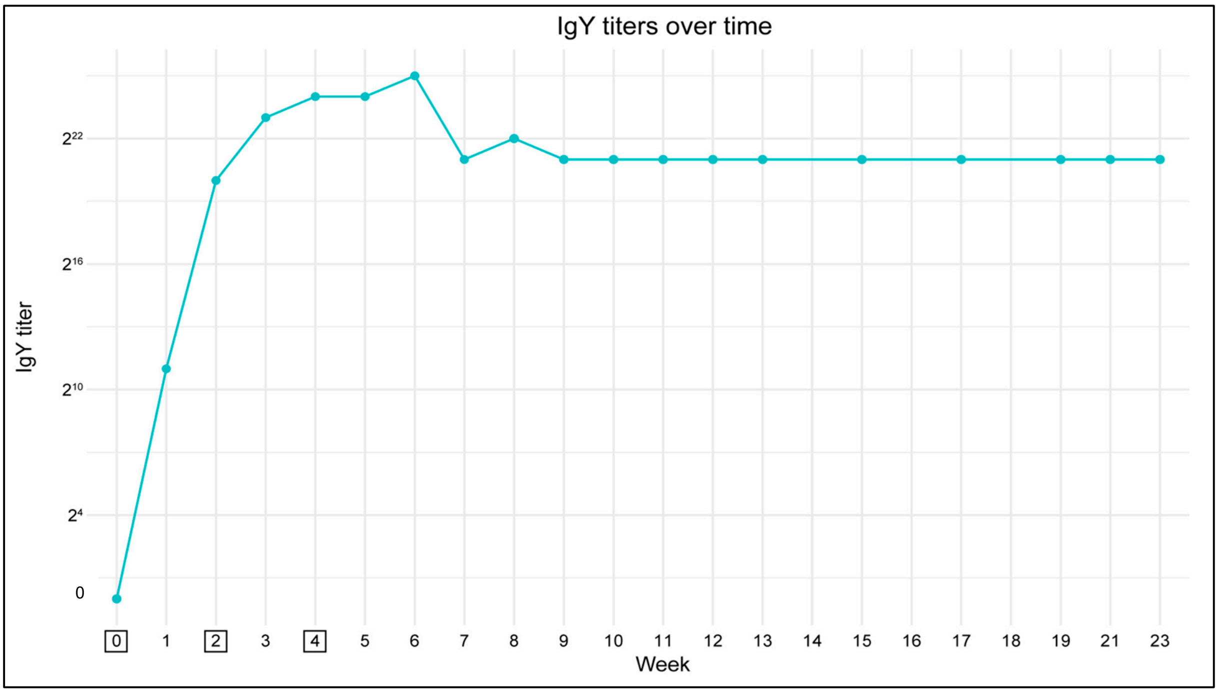

Figure S3. IgY Titers Over Time Following Immunizations. ELISA titers (log2) of IgY targeting HuNV GII.4 CHDC2094/1974 Virus-Like Particles (VLP) over 24 weeks are shown as a line plot. Immunizations were given at weeks 0, 2, and 4 (boxes). After seven weeks, HuNVLP IgY titers remained at or above 2^{-21} (1:2,097,152) through the end of the study period. The lower limit of detection (LLOD) for this assay was 2.1 times the OD_{450} value for the unimmunized control and defined as zero.

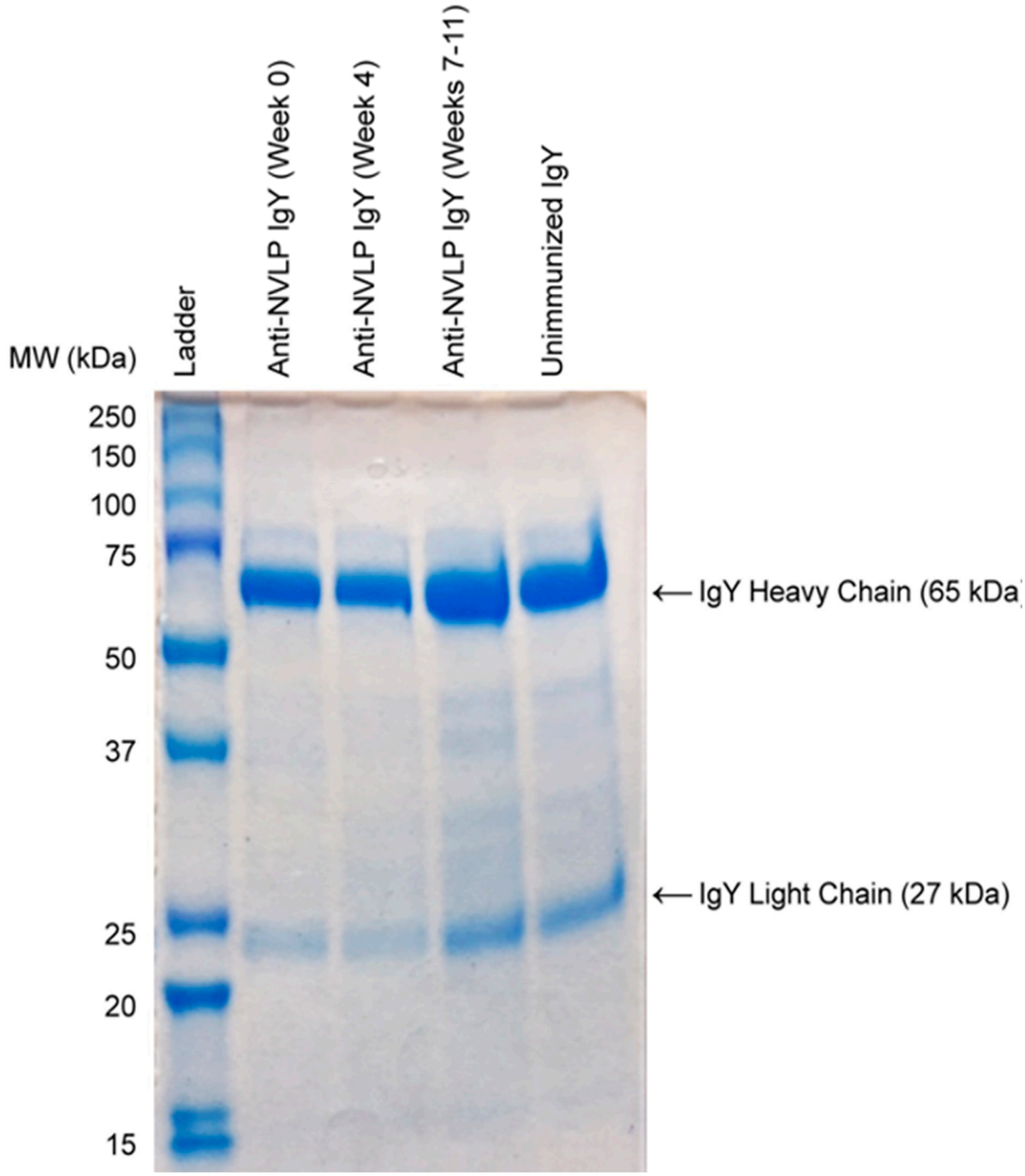

Figure S4. SDS-PAGE Analysis of anti-HuNoV IgY. SDS-Page Gel. Precision Plus Dual Color Pre-stained protein standard (10-250kDa; Bio-Rad, Hercules, CA, USA) was used as a molecular weight marker. Purified IgY from weeks 7-11 is pooled. IgY heavy and light chains show characteristic bands at 65 and 27 kDa, respectively. IgY from unimmunized hens was used as control.

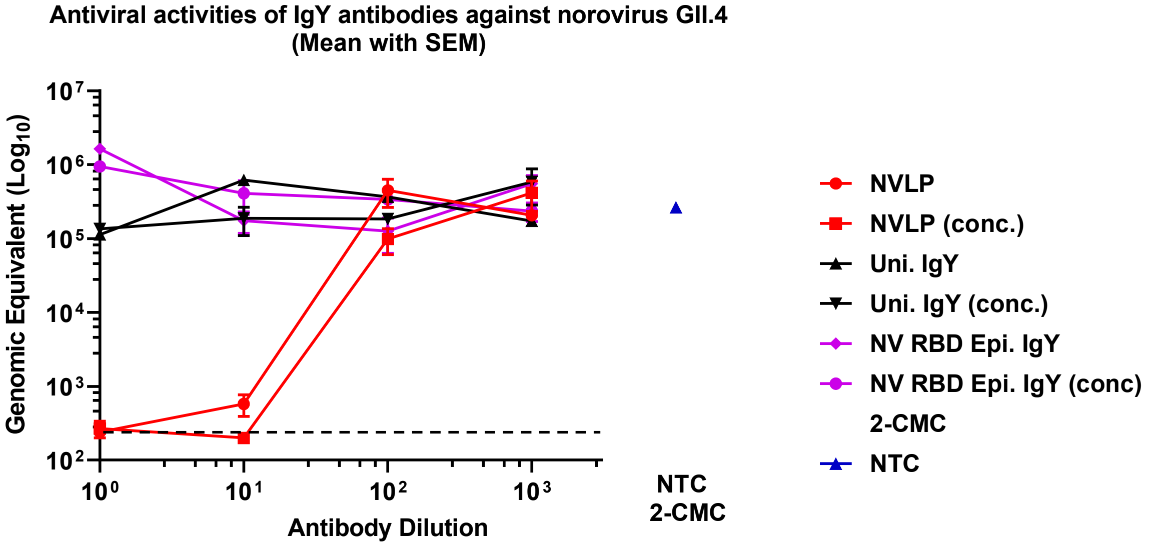

Figure S5. Antiviral activities of IgY antibodies against HuNoV GII.4 in HIE J2 cells. Antibodies were made 10-fold serial dilutions. Antibodies tested are listed in Table 1. 2-CMC (2'-C-methylcytidine) was used as a positive drug control and tested at a concentration of 200 μM. Dashed line indicates lower level of detection for HuNoV viral copies (200 genomic equivalents). NTC=non-treatment control (medium alone + virus) for virus growth in the cells. Limit of detection of the qRT-PCR 2 x 10^2 genome equivalent. "Conc." indicates IgY preparations concentrated 2-fold over the original "neat" material at 10 mg IgY/mL. Concentrated IgY conditions and anti-RBD (receptor binding domain) IgY were dropped from subsequent experiments.

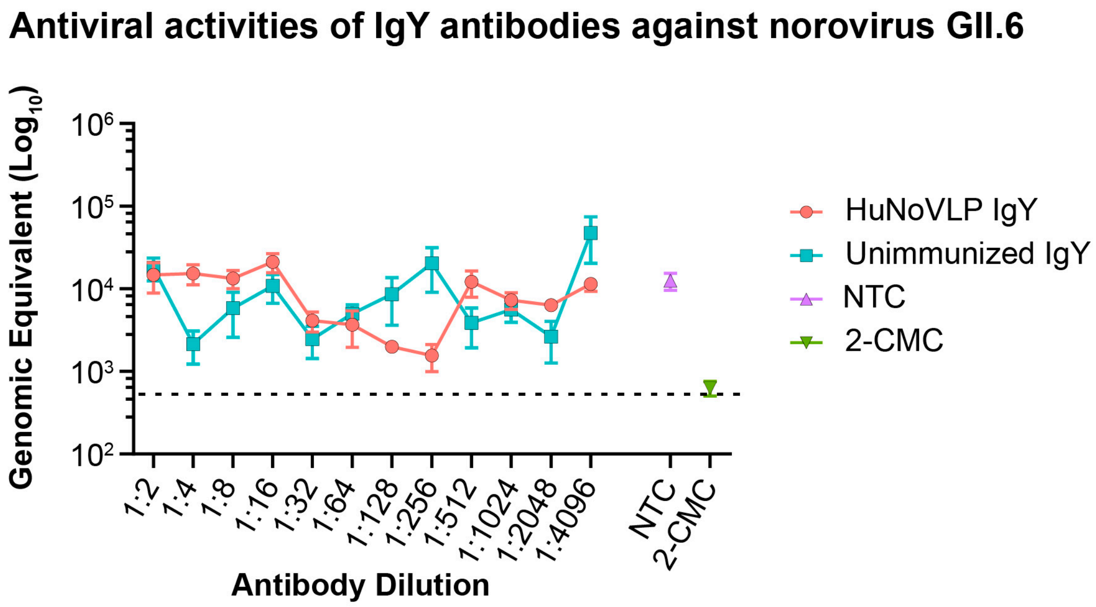

Figure S6. Antiviral activities of IgY antibodies against HuNoV GII.6 in HIE J2 cells. Antibodies targeting HuNoV GII.4 VLP (HuNoVLP IgY) were prepared in 2-fold dilutions, along with antibodies from unimmunized hens' eggs (Unimmunized IgY). Dashed line indicates lower level of detection for HuNoV viral copies (200 genomic equivalents). 2-CMC (2'-C-methylcytidine) was used as a positive drug control and tested at a concentration of 200 µM. NTC=non-treatment control (medium alone + virus) for virus growth in the cells.

Table S1: Coefficients of Variation (CV) for Each Experiment

A) HBGA Blocking Assay (Figure 1)

Test_Group	Dilution	mean	sd	cv
anti-NVLP	1 to 1024	0.079	0.00264575	0.03349052
anti-NVLP	1 to 128	0.04366667	0.00057735	0.01322176
anti-NVLP	1 to 2048	0.10266667	0.00208167	0.02027597
anti-NVLP	1 to 256	0.04533333	0.00416333	0.09183821
anti-NVLP	1 to 512	0.048	0.0052915	0.11023964
positive control	1 to 1024	0.15966667	0.0023094	0.01446389
positive control	1 to 128	0.15966667	0.0023094	0.01446389
positive control	1 to 2048	0.15966667	0.0023094	0.01446389
positive control	1 to 256	0.15966667	0.0023094	0.01446389
positive control	1 to 512	0.15966667	0.0023094	0.01446389
unimmunized	1 to 1024	0.15166667	0.00737112	0.04860076
unimmunized	1 to 128	0.135	0.00173205	0.01283001
unimmunized	1 to 2048	0.16233333	0.00321455	0.01980216
unimmunized	1 to 256	0.14766667	0.00737112	0.04991726
unimmunized	1 to 512	0.15466667	0.01193035	0.07713591

B) Viral Neutralization Assays – GII.4 (Figure 2A)

**Figure 2A**								
**Anti-HuNoV IgY**					**Unimmunized IgY**			
**Dilution**	**Mean**	**SD**	**CV**		**Dilution**	**Mean**	**SD**	**CV**
1:2	200	0.000	**0.000**		1:2	494826.8	86029.26	**0.174**
1:4	219.25	38.500	**0.176**		1:4	413966	262183.8	**0.633**
1:8	200	0.000	**0.000**		1:8	525246.3	102370.1	**0.195**
1:16	200	0.000	**0.000**		1:16	132590	61611.78	**0.465**
1:32	397.25	239.451	**0.603**		1:32	517473.8	18962.12	**0.037**
1:64	588.25	95.925	**0.163**		1:64	416350.5	204018.5	**0.490**
1:128	44623.25	50100.819	**1.123**		1:128	219343.8	132181.2	**0.603**
1:256	354086.8	344929.594	**0.974**		1:256	533215	32258.09	**0.060**
1:512	894450	843208.666	**0.943**		1:512	427616.5	254775.6	**0.596**
1:1024	478038.5	179205.021	**0.375**		1:1024	865036.9	179919.8	**0.208**
1:2048	851238	337619.434	**0.397**		1:2048	609085.1	304271.4	**0.500**
1:4096	51249.25	26825.643	**0.523**		1:4096	370998.7	331818.1	**0.894**

C) Viral Neutralization Assays – GII.4 (Figure 2B)

Figure 2B								
Anti-HuNoV IgY				Unimmunized IgY				
Dilution	Mean	SD	CV		Dilution	Mean	SD	CV
1:64	206	16.971	0.082		1:64	354753.8	248875.318	0.702
1:128	515	126.636	0.246		1:128	173418.167	147141.664	0.848
1:256	12134.75	14242.380	1.174		1:256	45045.8	46903.303	1.041
1:512	82729.75	77981.536	0.943		1:512	531833.833	625388.319	1.176
1:1024	72735.75	56488.241	0.777		1:1024	295407.6	225973.766	0.765
1:2048	421439.75	578349.101	1.372		1:2048	464040	656774.648	1.415
1:4096	373056	167943.867	0.450		1:4096	174887	69837.262	0.399

D) Viral Neutralization Assays – GII.6 (Figure S6)

Figure S6								
Anti-HuNoV IgY					Unimmunized IgY			
Dilution	Mean	SD	CV		Dilution	Mean	SD	CV
1:2	14766	16726	1.133		1:2	17981	13472	0.749
1:4	15358	11849	0.771		1:4	2154	2269	1.054
1:8	13379	9399	0.703		1:8	5816	7256	1.248
1:16	21153	15524	0.734		1:16	10843	10129	0.934
1:32	4115	2768	0.673		1:32	2472	2340	0.946
1:64	3673	3420	0.931		1:64	4998	3447	0.690
1:128	1985	187	0.094		1:128	8613	11185	1.299
1:256	1553	1118	0.720		1:256	20266	27449	1.354
1:512	12169	10412	0.856		1:512	3887	4817	1.239
1:1024	7297	4647	0.637		1:1024	5613	4084	0.728
1:2048	6346	1275	0.201		1:2048	2641	3076	1.164
1:4096	11481	6249	0.544		1:4096	47305	65883	1.393
